# (2-{[2-(4-Chlorophenoxy)-1-oxido­ethyl­idene-κ*O* ^1^]hydrazono­methyl}­phenol­ato-κ^2^ *N* ^1^,*O*)(1*H*-imidazole-κ*N* ^3^)nickel(II)

**DOI:** 10.1107/S1600536808028171

**Published:** 2008-09-06

**Authors:** Xiao-Hua Chen

**Affiliations:** a College of Chemistry and Materials Science, Fujian Normal University, Fuzhou, Fujian 350007, People’s Republic of China

## Abstract

In the title complex, [Ni(C_15_H_11_ClN_2_O_3_)(C_3_H_4_N_2_)], the Ni^II^ ion is coordinated by a phenolate O, hydrazine N and carbonyl O atom from the hydrazone ligand and by an N atom from the imidazole mol­ecule, forming a distorted square-planar geometry. Inter­molecular N—H⋯N hydrogen bonds link neighboring molecules into extended chains parallel to [100].

## Related literature

For general background, see: Liu & Gao (1998 ▶); Ma *et al.* (1989 ▶); Sur *et al.* (1993 ▶); Sun *et al.* (2005 ▶). For related structures, see: Chen & Liu (2006 ▶).
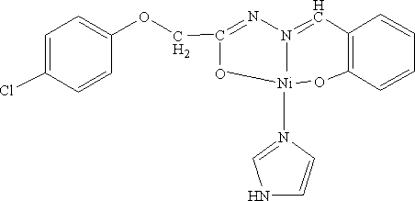


## Experimental

### 

#### Crystal data

[Ni(C_15_H_11_ClN_2_O_3_)(C_3_H_4_N_2_)]
*M* *_r_* = 429.50
Orthorhombic, 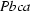

*a* = 18.745 (5) Å
*b* = 6.6054 (14) Å
*c* = 29.230 (8) Å
*V* = 3619.2 (16) Å^3^
*Z* = 8
Mo *K*α radiation
μ = 1.25 mm^−1^
*T* = 293 (2) K
0.65 × 0.21 × 0.15 mm

#### Data collection

Rigaku R-AXIS RAPID diffractometer
Absorption correction: multi-scan (*TEXRAY*; Molecular Structure Corporation, 1999 ▶) *T*_min_ = 0.498, *T*_max_ = 0.835
29384 measured reflections
4146 independent reflections
2947 reflections with *I* > 2σ(*I*)
*R*_int_ = 0.058

#### Refinement

*R*[*F* ^2^ > 2σ(*F* ^2^)] = 0.035
*wR*(*F* ^2^) = 0.087
*S* = 0.96
4146 reflections
244 parameters
H-atom parameters constrained
Δρ_max_ = 0.40 e Å^−3^
Δρ_min_ = −0.30 e Å^−3^

### 

Data collection: *TEXRAY* (Molecular Structure Corporation, 1999 ▶); cell refinement: *TEXRAY*; data reduction: *TEXSAN* (Mol­ec­ular Structure Corporation, 1999 ▶); program(s) used to solve structure: *SHELXS97* (Sheldrick, 2008 ▶); program(s) used to refine structure: *SHELXL97* (Sheldrick, 2008 ▶); molecular graphics: *ORTEX* (McArdle, 1995 ▶); software used to prepare material for publication: *SHELXL97*.

## Supplementary Material

Crystal structure: contains datablocks I, global. DOI: 10.1107/S1600536808028171/bv2105sup1.cif

Structure factors: contains datablocks I. DOI: 10.1107/S1600536808028171/bv2105Isup2.hkl

Additional supplementary materials:  crystallographic information; 3D view; checkCIF report

## Figures and Tables

**Table 1:** Hydrogen-bond geometry (Å, °)

*D*—H⋯*A*	*D*—H	H⋯*A*	*D*⋯*A*	*D*—H⋯*A*
N4—H4*A*⋯N2^i^	0.86	2.06	2.916 (3)	172

Symmetry code: (i) 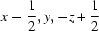.

**Fig. 1.**
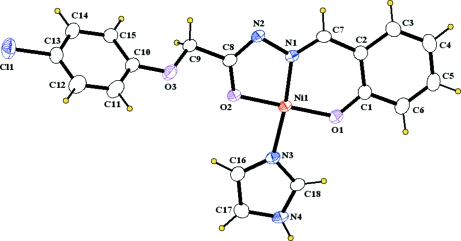
The structure of (I), showing 30% probability displacement ellipsoids and the atom-numbering scheme.

**Fig. 2.**
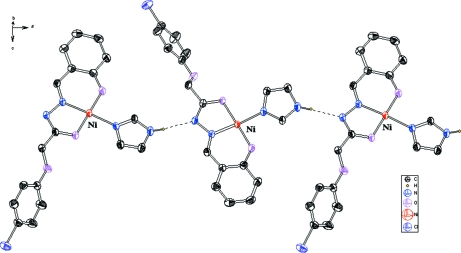
Extended chain structure of (I).

## supplementary crystallographic information

### Comment

Hydrazones are of interest owing to their capacity for chelating to transition (Sur *et al.*, 1993; Sun *et al.*, 2005), lanthanide (Ma *et al.*, 1989) and main group (Liu & Gao 1998) metals. Here we report the crystal structure of the title complex, (I) (Fig. 1). The Ni(II) ion exists in a square-planar N_2_O_2_ coordination geometry defined by the phenolate O1, hydrazine N1, and carbonyl O2 atom of the hydrazone ligand, and N3 atom from the 1*H*-imidazole molecule. The hydrazone ligand in the title complex is distorted, the dihedral angle between the two phenyl rings being 50.56 (9)°. The corresponding dihedral angle is about 87° in the complex [Co(C_15_H_12_N_2_O_3_)(C_5_H_7_O_2_)(C_3_H_7_NO)] (Chen & Liu, 2006) with the ligand of salicylaldehyde phenoxyacylhydrazone. In the title complex, an extended one-dimensional chain structure is formed *via* intermolecular hydrogen bonds between the 1*H*-imidazole N—H groups and the uncoordinated N-atom of the hydrazone ligand (Fig. 2). The N4···N2(A) distance and the N4—H···N2(A) angle are 2.916 (3) Å and 171.92°, respectively [Symmetry code: (A) *x* - 1/2, *y*, -*z* + 1/2].

### Experimental

The hydrazone ligand was prepared by the reaction of salicylaldehyde and 4-chloro-phenoxyacetylhydrazine in a molar ratio of 1:1 under reflux in ethanol for 2 h. The yellow product obtained on cooling was recrystallized from methanol. Salicylaldehyde 4-chloro-phenoxyacetylhydrazone (1 mmol), [Ni(OAc)_2_].4H_2_O (1 mmol), imidazole (1 mmol), N,N-dimethylformamide (5 ml), and methanol (10 ml) were stirred for 2 h. The solution was filtered and allowed to stand at room temperature for one week, and red crystals of complex (I) were obtained.

### Refinement

All H atoms were placed in idealized positions and treated as riding with N—H = 0.86 Å, C—H = 0.93–0.97Å and *U*_iso_(H) = 1.2*U*_eq_(C/N).

### Crystal data

**Table:**

[Ni(C_15_H_11_ClN_2_O_3_)(C_3_H_4_N_2_)]	*F*(000) = 1760
*M**_r_* = 429.50	*D*_x_ = 1.576 Mg m^−^^3^
Orthorhombic, *P**b**c**a*	Mo *K*α radiation, λ = 0.71073 Å
Hall symbol: -P 2ac 2ab	Cell parameters from 2947 reflections
*a* = 18.745 (5) Å	θ = 3.0–27.5°
*b* = 6.6054 (14) Å	µ = 1.25 mm^−^^1^
*c* = 29.230 (8) Å	*T* = 293 K
*V* = 3619.2 (16) Å^3^	Prism, red
*Z* = 8	0.65 × 0.21 × 0.15 mm

### Data collection

**Table:**

Rigaku R-AXIS RAPID diffractometer	4146 independent reflections
Radiation source: fine-focus sealed tube	2947 reflections with *I* > 2σ(*I*)
graphite	*R*_int_ = 0.058
ω scans	θ_max_ = 27.5°, θ_min_ = 3.0°
Absorption correction: multi-scan (*TEXRAY*; Molecular Structure Corporation, 1999)	*h* = −24→24
*T*_min_ = 0.498, *T*_max_ = 0.835	*k* = −8→8
29384 measured reflections	*l* = −37→37

### Refinement

**Table:**

Refinement on *F*^2^	Primary atom site location: structure-invariant direct methods
Least-squares matrix: full	Secondary atom site location: difference Fourier map
*R*[*F*^2^ > 2σ(*F*^2^)] = 0.035	Hydrogen site location: inferred from neighbouring sites
*wR*(*F*^2^) = 0.087	H-atom parameters constrained
*S* = 0.96	*w* = 1/[σ^2^(*F*_o_^2^) + (0.0475*P*)^2^] where *P* = (*F*_o_^2^ + 2*F*_c_^2^)/3
4146 reflections	(Δ/σ)_max_ = 0.001
244 parameters	Δρ_max_ = 0.40 e Å^−^^3^
0 restraints	Δρ_min_ = −0.30 e Å^−^^3^

### Special details

**Table:**

Geometry. All e.s.d.'s (except the e.s.d. in the dihedral angle between two l.s. planes) are estimated using the full covariance matrix. The cell e.s.d.'s are taken into account individually in the estimation of e.s.d.'s in distances, angles and torsion angles; correlations between e.s.d.'s in cell parameters are only used when they are defined by crystal symmetry. An approximate (isotropic) treatment of cell e.s.d.'s is used for estimating e.s.d.'s involving l.s. planes.
Refinement. Refinement of *F*^2^ against ALL reflections. The weighted *R*-factor *wR* and goodness of fit *S* are based on *F*^2^, conventional *R*-factors *R* are based on *F*, with *F* set to zero for negative *F*^2^. The threshold expression of *F*^2^ > σ(*F*^2^) is used only for calculating *R*-factors(gt) *etc*. and is not relevant to the choice of reflections for refinement. *R*-factors based on *F*^2^ are statistically about twice as large as those based on *F*, and *R*- factors based on ALL data will be even larger.

### Fractional atomic coordinates and isotropic or equivalent isotropic displacement parameters (Å^2^)

**Table:**

	*x*	*y*	*z*	*U*_iso_*/*U*_eq_	
Ni1	0.160819 (13)	0.65045 (4)	0.262178 (8)	0.03514 (10)	
O1	0.11416 (8)	0.6681 (2)	0.31655 (5)	0.0519 (4)	
O2	0.20944 (7)	0.63914 (19)	0.20727 (4)	0.0397 (3)	
O3	0.30335 (9)	0.7513 (2)	0.14068 (5)	0.0524 (4)	
N1	0.25009 (9)	0.6404 (2)	0.28711 (5)	0.0344 (3)	
N2	0.30584 (9)	0.6336 (2)	0.25500 (6)	0.0381 (4)	
N3	0.07133 (9)	0.6479 (2)	0.23119 (6)	0.0403 (4)	
N4	−0.04380 (10)	0.6469 (3)	0.21963 (6)	0.0489 (4)	
H4A	−0.0889	0.6519	0.2250	0.059*	
Cl1	0.44937 (4)	0.82246 (10)	−0.03563 (2)	0.0718 (2)	
C1	0.14251 (12)	0.6600 (3)	0.35761 (7)	0.0433 (5)	
C2	0.21615 (12)	0.6403 (3)	0.36618 (7)	0.0393 (4)	
C3	0.24043 (14)	0.6287 (3)	0.41153 (7)	0.0508 (6)	
H3A	0.2890	0.6143	0.4171	0.061*	
C4	0.19446 (16)	0.6382 (3)	0.44779 (8)	0.0580 (6)	
H4B	0.2114	0.6285	0.4776	0.070*	
C5	0.12234 (16)	0.6625 (4)	0.43930 (8)	0.0589 (6)	
H5A	0.0908	0.6713	0.4638	0.071*	
C6	0.09647 (14)	0.6737 (4)	0.39544 (8)	0.0575 (6)	
H6A	0.0478	0.6906	0.3907	0.069*	
C7	0.26712 (11)	0.6330 (3)	0.32997 (7)	0.0396 (4)	
H7A	0.3151	0.6224	0.3376	0.048*	
C8	0.27699 (11)	0.6312 (3)	0.21432 (7)	0.0376 (4)	
C9	0.32487 (11)	0.6078 (3)	0.17375 (7)	0.0430 (5)	
H9A	0.3741	0.6312	0.1825	0.052*	
H9B	0.3210	0.4719	0.1614	0.052*	
C10	0.33961 (11)	0.7564 (3)	0.10033 (7)	0.0432 (5)	
C11	0.32289 (16)	0.9148 (4)	0.07150 (9)	0.0694 (8)	
H11A	0.2890	1.0099	0.0804	0.083*	
C12	0.35588 (16)	0.9335 (4)	0.02962 (9)	0.0709 (8)	
H12A	0.3440	1.0399	0.0102	0.085*	
C13	0.40604 (13)	0.7950 (3)	0.01670 (7)	0.0505 (5)	
C14	0.42323 (14)	0.6372 (3)	0.04485 (7)	0.0533 (6)	
H14A	0.4573	0.5431	0.0357	0.064*	
C15	0.39005 (13)	0.6170 (3)	0.08685 (7)	0.0481 (5)	
H15A	0.4018	0.5094	0.1060	0.058*	
C16	0.05887 (13)	0.6245 (4)	0.18536 (8)	0.0545 (6)	
H16A	0.0938	0.6107	0.1630	0.065*	
C17	−0.01198 (13)	0.6245 (4)	0.17777 (8)	0.0619 (7)	
H17A	−0.0348	0.6117	0.1497	0.074*	
C18	0.00820 (11)	0.6595 (3)	0.25023 (8)	0.0418 (5)	
H18A	0.0008	0.6748	0.2815	0.050*	

### Atomic displacement parameters (Å^2^)

**Table:**

	*U*^11^	*U*^22^	*U*^33^	*U*^12^	*U*^13^	*U*^23^
Ni1	0.02247 (14)	0.04455 (16)	0.03838 (15)	−0.00093 (10)	0.00212 (10)	0.00103 (11)
O1	0.0303 (8)	0.0837 (11)	0.0416 (8)	0.0012 (8)	0.0047 (6)	0.0011 (7)
O2	0.0259 (7)	0.0523 (8)	0.0408 (7)	−0.0004 (6)	0.0013 (6)	0.0006 (6)
O3	0.0488 (10)	0.0576 (9)	0.0507 (8)	0.0156 (8)	0.0168 (7)	0.0097 (7)
N1	0.0259 (8)	0.0354 (8)	0.0419 (9)	0.0007 (7)	0.0018 (7)	0.0000 (7)
N2	0.0249 (8)	0.0471 (9)	0.0422 (9)	0.0004 (7)	0.0043 (7)	0.0010 (7)
N3	0.0268 (9)	0.0488 (9)	0.0453 (9)	−0.0028 (7)	0.0023 (7)	−0.0002 (8)
N4	0.0238 (9)	0.0643 (11)	0.0585 (11)	0.0004 (8)	−0.0006 (8)	0.0004 (9)
Cl1	0.0848 (5)	0.0815 (4)	0.0491 (3)	0.0023 (4)	0.0225 (3)	0.0045 (3)
C1	0.0442 (13)	0.0457 (11)	0.0400 (10)	−0.0037 (10)	0.0040 (9)	0.0005 (9)
C2	0.0423 (12)	0.0345 (9)	0.0413 (10)	0.0001 (9)	0.0003 (9)	0.0005 (8)
C3	0.0533 (15)	0.0526 (13)	0.0467 (12)	0.0052 (11)	−0.0053 (11)	−0.0010 (10)
C4	0.0749 (19)	0.0589 (14)	0.0401 (11)	0.0025 (14)	0.0010 (12)	0.0006 (10)
C5	0.0646 (18)	0.0688 (15)	0.0433 (12)	−0.0019 (13)	0.0152 (12)	−0.0017 (11)
C6	0.0441 (14)	0.0787 (16)	0.0497 (12)	−0.0033 (12)	0.0116 (11)	0.0007 (12)
C7	0.0305 (11)	0.0434 (10)	0.0448 (11)	0.0030 (9)	−0.0038 (9)	0.0021 (9)
C8	0.0282 (10)	0.0399 (10)	0.0448 (11)	0.0005 (8)	0.0034 (9)	0.0026 (9)
C9	0.0302 (11)	0.0569 (12)	0.0418 (11)	0.0031 (9)	0.0044 (9)	0.0037 (10)
C10	0.0353 (12)	0.0492 (11)	0.0450 (11)	−0.0007 (9)	0.0067 (9)	0.0012 (10)
C11	0.0705 (19)	0.0652 (15)	0.0725 (17)	0.0248 (14)	0.0267 (15)	0.0204 (14)
C12	0.076 (2)	0.0674 (15)	0.0692 (16)	0.0195 (15)	0.0218 (15)	0.0269 (14)
C13	0.0502 (14)	0.0613 (13)	0.0400 (10)	−0.0037 (11)	0.0074 (10)	0.0007 (10)
C14	0.0522 (14)	0.0614 (14)	0.0463 (12)	0.0104 (12)	0.0085 (11)	−0.0039 (10)
C15	0.0456 (13)	0.0551 (13)	0.0436 (11)	0.0105 (11)	0.0018 (10)	0.0046 (10)
C16	0.0335 (12)	0.0849 (17)	0.0452 (11)	−0.0035 (12)	0.0035 (10)	−0.0045 (11)
C17	0.0363 (13)	0.100 (2)	0.0490 (13)	−0.0063 (13)	−0.0056 (10)	−0.0039 (13)
C18	0.0267 (10)	0.0506 (11)	0.0480 (11)	0.0006 (9)	0.0016 (9)	−0.0035 (10)

### Geometric parameters (Å, °)

**Table:**

Ni1—O1	1.8178 (14)	C4—C5	1.384 (4)
Ni1—N1	1.8264 (17)	C4—H4B	0.9300
Ni1—O2	1.8473 (14)	C5—C6	1.373 (3)
Ni1—N3	1.9064 (18)	C5—H5A	0.9300
O1—C1	1.314 (3)	C6—H6A	0.9300
O2—C8	1.284 (2)	C7—H7A	0.9300
O3—C10	1.362 (2)	C8—C9	1.495 (3)
O3—C9	1.413 (2)	C9—H9A	0.9700
N1—C7	1.294 (3)	C9—H9B	0.9700
N1—N2	1.405 (2)	C10—C15	1.377 (3)
N2—C8	1.307 (3)	C10—C11	1.380 (3)
N3—C18	1.310 (3)	C11—C12	1.377 (3)
N3—C16	1.368 (3)	C11—H11A	0.9300
N4—C18	1.326 (3)	C12—C13	1.365 (4)
N4—C17	1.369 (3)	C12—H12A	0.9300
N4—H4A	0.8600	C13—C14	1.367 (3)
Cl1—C13	1.741 (2)	C14—C15	1.383 (3)
C1—C6	1.406 (3)	C14—H14A	0.9300
C1—C2	1.409 (3)	C15—H15A	0.9300
C2—C3	1.404 (3)	C16—C17	1.347 (3)
C2—C7	1.427 (3)	C16—H16A	0.9300
C3—C4	1.367 (3)	C17—H17A	0.9300
C3—H3A	0.9300	C18—H18A	0.9300
			
O1—Ni1—N1	95.41 (7)	N1—C7—H7A	118.3
O1—Ni1—O2	178.45 (7)	C2—C7—H7A	118.3
N1—Ni1—O2	83.87 (7)	O2—C8—N2	123.63 (19)
O1—Ni1—N3	89.58 (7)	O2—C8—C9	117.97 (18)
N1—Ni1—N3	174.52 (7)	N2—C8—C9	118.34 (18)
O2—Ni1—N3	91.20 (7)	O3—C9—C8	107.58 (16)
C1—O1—Ni1	126.98 (15)	O3—C9—H9A	110.2
C8—O2—Ni1	110.41 (13)	C8—C9—H9A	110.2
C10—O3—C9	117.83 (16)	O3—C9—H9B	110.2
C7—N1—N2	117.51 (17)	C8—C9—H9B	110.2
C7—N1—Ni1	127.87 (14)	H9A—C9—H9B	108.5
N2—N1—Ni1	114.59 (12)	O3—C10—C15	125.01 (19)
C8—N2—N1	107.48 (16)	O3—C10—C11	115.7 (2)
C18—N3—C16	105.56 (19)	C15—C10—C11	119.2 (2)
C18—N3—Ni1	126.33 (15)	C12—C11—C10	120.6 (2)
C16—N3—Ni1	128.04 (15)	C12—C11—H11A	119.7
C18—N4—C17	106.83 (19)	C10—C11—H11A	119.7
C18—N4—H4A	126.6	C13—C12—C11	119.7 (2)
C17—N4—H4A	126.6	C13—C12—H12A	120.1
O1—C1—C6	117.9 (2)	C11—C12—H12A	120.1
O1—C1—C2	124.22 (19)	C12—C13—C14	120.5 (2)
C6—C1—C2	117.9 (2)	C12—C13—Cl1	119.65 (18)
C3—C2—C1	119.4 (2)	C14—C13—Cl1	119.89 (19)
C3—C2—C7	118.8 (2)	C13—C14—C15	120.1 (2)
C1—C2—C7	121.83 (19)	C13—C14—H14A	119.9
C4—C3—C2	121.7 (2)	C15—C14—H14A	119.9
C4—C3—H3A	119.2	C10—C15—C14	119.9 (2)
C2—C3—H3A	119.2	C10—C15—H15A	120.1
C3—C4—C5	118.8 (2)	C14—C15—H15A	120.1
C3—C4—H4B	120.6	C17—C16—N3	109.2 (2)
C5—C4—H4B	120.6	C17—C16—H16A	125.4
C6—C5—C4	121.3 (2)	N3—C16—H16A	125.4
C6—C5—H5A	119.4	C16—C17—N4	106.4 (2)
C4—C5—H5A	119.4	C16—C17—H17A	126.8
C5—C6—C1	121.0 (2)	N4—C17—H17A	126.8
C5—C6—H6A	119.5	N3—C18—N4	112.0 (2)
C1—C6—H6A	119.5	N3—C18—H18A	124.0
N1—C7—C2	123.49 (19)	N4—C18—H18A	124.0
			
N1—Ni1—O1—C1	−3.95 (18)	C2—C1—C6—C5	−1.8 (3)
O2—Ni1—O1—C1	−66 (2)	N2—N1—C7—C2	179.46 (15)
N3—Ni1—O1—C1	173.78 (18)	Ni1—N1—C7—C2	−2.8 (3)
O1—Ni1—O2—C8	62 (2)	C3—C2—C7—N1	179.26 (18)
N1—Ni1—O2—C8	−0.19 (13)	C1—C2—C7—N1	−1.2 (3)
N3—Ni1—O2—C8	−177.80 (13)	Ni1—O2—C8—N2	−0.8 (2)
O1—Ni1—N1—C7	4.62 (17)	Ni1—O2—C8—C9	176.13 (13)
O2—Ni1—N1—C7	−176.76 (16)	N1—N2—C8—O2	1.6 (3)
N3—Ni1—N1—C7	−150.9 (7)	N1—N2—C8—C9	−175.36 (16)
O1—Ni1—N1—N2	−177.59 (11)	C10—O3—C9—C8	−179.55 (18)
O2—Ni1—N1—N2	1.03 (11)	O2—C8—C9—O3	48.3 (2)
N3—Ni1—N1—N2	26.9 (8)	N2—C8—C9—O3	−134.64 (19)
C7—N1—N2—C8	176.44 (17)	C9—O3—C10—C15	8.3 (3)
Ni1—N1—N2—C8	−1.59 (17)	C9—O3—C10—C11	−172.1 (2)
O1—Ni1—N3—C18	0.86 (18)	O3—C10—C11—C12	−179.4 (3)
N1—Ni1—N3—C18	156.5 (6)	C15—C10—C11—C12	0.3 (4)
O2—Ni1—N3—C18	−177.80 (17)	C10—C11—C12—C13	−0.6 (5)
O1—Ni1—N3—C16	−175.8 (2)	C11—C12—C13—C14	0.6 (4)
N1—Ni1—N3—C16	−20.2 (8)	C11—C12—C13—Cl1	−178.4 (2)
O2—Ni1—N3—C16	5.55 (19)	C12—C13—C14—C15	−0.3 (4)
Ni1—O1—C1—C6	−179.05 (14)	Cl1—C13—C14—C15	178.68 (19)
Ni1—O1—C1—C2	1.5 (3)	O3—C10—C15—C14	179.7 (2)
O1—C1—C2—C3	−178.61 (18)	C11—C10—C15—C14	0.0 (4)
C6—C1—C2—C3	2.0 (3)	C13—C14—C15—C10	0.0 (4)
O1—C1—C2—C7	1.8 (3)	C18—N3—C16—C17	0.6 (3)
C6—C1—C2—C7	−177.61 (18)	Ni1—N3—C16—C17	177.80 (16)
C1—C2—C3—C4	−0.7 (3)	N3—C16—C17—N4	−0.4 (3)
C7—C2—C3—C4	178.91 (19)	C18—N4—C17—C16	0.1 (3)
C2—C3—C4—C5	−0.9 (3)	C16—N3—C18—N4	−0.6 (2)
C3—C4—C5—C6	1.1 (4)	Ni1—N3—C18—N4	−177.83 (13)
C4—C5—C6—C1	0.3 (4)	C17—N4—C18—N3	0.3 (2)
O1—C1—C6—C5	178.8 (2)		

### Hydrogen-bond geometry (Å, °)

**Table:**

*D*—H···*A*	*D*—H	H···*A*	*D*···*A*	*D*—H···*A*
N4—H4A···N2^i^	0.86	2.06	2.916 (3)	172

Symmetry codes: (i) *x*−1/2, *y*, −*z*+1/2.
